# Delayed seroconversion to STLV-1 infection is associated with mutations in the pol and rex genes

**DOI:** 10.1186/1743-422X-10-282

**Published:** 2013-09-11

**Authors:** Syamalima Dube, Nitin Saksena, Timothy Spicer, Jayne Healey, Patricia Benz, Dipak K Dube, Bernard J Poiesz

**Affiliations:** 1 Division of Hematology/Oncology, Department of Medicine, State University of New York, Upstate Medical University, 750 East Adams Street, Syracuse, NY 13210, USA; 2 Retroviral Genetics Division, Center for Virus Research, Westmead Millennium Institute, University of Sydney, Westmead NSW 2145, Sydney, Australia

## Abstract

**Background:**

Simian T-cell lymphoma/leukemia virus-1 (STLV-1) infection of non-human primates can serve as a model for human T-cell lymphoma/leukemia virus infection.

**Methods:**

Two tantalus and 2 *patas* monkeys were transfused with intraspecies whole blood infected with STLV-1. Infection was determined by ELISA, western blot and DNA PCR analyses. The entire genome of the STLV-1 Tan 90 strain and some of the STVL-1 Pat74 strain were amplified using over-lapping primer-pairs and subsequently sequenced.

**Results:**

Followup studies conducted over 2 years indicated that all 4 monkeys remained healthy despite being infected with STLV-1, as determined by PCR, cloning and sequencing analyses. ELISA and Western blot analyses indicated that both *patas* monkeys seroconverted within 2 months of transfusion, while one tantalus monkey required one year to seroconvert and the other never fully seroconverted. The tantalus monkey which never fully seroconverted, failed to react to HTLV-1 p24 Gag antigen. Sequence analyses indicated that, while unique, the deduced p24 Gag amino acid sequence of the STLV-1 Tan 90 strain used for infection was still highly homologous to the HTLV-1 p24 Gag amino acids present in the ELISA and WB assays. However, a mutation in the *pol* sequence of STLV-1 Tan 90 encoded a putative stop codon, while a common deletion in the *pol/rex* regulatory gene causes significant changes in the Pol, and p27 Rex proteins. These same mutations were also observed in the viral DNA of both recipient infected tantalus monkeys and were not present in the STLV-1 Pat 74 strain.

**Conclusion:**

Our data suggest that seroconversion to STLV-1 infection may be prolonged due to the above mutations, and that compensatory molecular events must have occurred to allow for virus transmission.

## Introduction

The primate T-cell lymphoma/leukemia viruses (PTLV) are comprised of at least four, and possibly six, distinct species that infect both simians (STLV) and/or humans (HTLV) [1,2]. Relative to other primate retroviruses (e.g. HIV, SIV), PTLV transmission is often characterized by slow or indeterminate seroconversion [3,4]. HTLV-1 is associated with a variety of clinical disorders including T-cell lymphomas and leukemias, neurodegenerative disease, polymyositis, arthritis and uveitis [5]. STLV-1 has also been shown to cause T-cell lymphomas and leukemias [6-8]. Hence, STLV-1 infection of non-human primates could serve as a model for human PTLV infection, seroconversion, and disease pathogenesis.

In the past, we described that STLV-I infection was endemic among *Chlorocebus* (African green monkeys) and *Erythrocebus patas* (African red monkeys) in Central African Republic [9,10]. Two unique strains, STLV-1 Tan 90 and STLV-1 Pat 74 from a *Chlorocebus* tantalus and a *Erthrocebus patas*, respectively, were identified. These strains diverge from the prototype Japanese HTLV-I (ATK) isolate by 7.1% and 5%, respectively, and from each other by 9.3%. Herein, we describe experimental intraspecies transmission of these two strains resulting in varied seroconversion patterns. An extensive sequence analysis was conducted on both strains to seek an explanation(s) for the observed differences in seroconversion.

## Results

During the entire two year observation period all three tantalus and three *patas* monkeys remained healthy. Their complete blood counts, CD4 and CD8 counts remained stable and within normal limits (data not shown). None of the animals developed clinical signs of a PTLV- associated disease.

**Figure 1:**
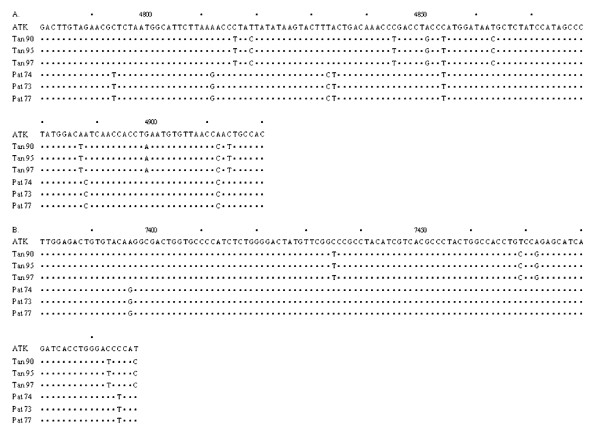
**Alignment of *pol *(A) and *pX *(B) sequences from tantalus or *patas *monkeys infected with either STLV-I (Tan 90) or STLV-I (Pat 74).** Base changes from the prototypic HTLV-I (ATK) sequence are shown. The last digit of the number is above the corresponding base.

The serological and PCR analyses on the tantalus and *patas* monkeys transfused with whole blood from Tan 90 and Pat 74, are shown in Table 1. As can be seen, following transfusion, all monkeys were ultimately shown to be infected by PCR analyses for the STLV-1 *pol* and *pX* genes (Table 1). Sequence analyses of the amplified DNA indicated that the tantalus and *patas* monkeys were infected with the STLV-I isolates that they had been inoculated with; i.e. STLV-1 Tan90 and STLV-1 Pat 74, respectively (Figure 1).

**Table 1:** **Chronology of serological (ELISA** &**WB) and PCR analyses of monkeys experimentally infected with STLV-1 Tan 90 or STLV-1 Pat 74**

**Target monkey**	**SIV**	**Inoculumn**	**Months post exposure**	**ELISA**	**WB**											
					Status *	**rgp21**	**p19**	**p24**	**p26**	**p28**	**p32**	**p36**	**gp46**	**p55**	**rgp46**	**PCR**^**▲**^
Tan 95	+	STLV-1 Tan 90	0	-	-	-	-	-	-	-	-	-	-	-	-	-
			2	-	-	-	-	-	-	-	-	-	-	-	-	-
			4	-	-	-	-	-	-	-	-	-	-	-	-	+
			11	-	I	-	++	-	+	+	-	+	-	-	-	+
			12	+	I	+	++	-	+	+	+	+	-	+	+++	+
			24	+	I	+	++	-	+	+	+	+	-	+	+++	+
Tan 97	-	STLV-1 Tan 90	0	-	-	-	-	-	-	-	-	-	-	-	-	-
			2	-	-	-	-	-	-	-	-	-	-	-	-	-
			4	-	-	-	-	-	-	-	-	-	-	-	-	+
			11	-	+	+	+++	+++	++	+++	+	++	+	+	+	+
			12	+	+	+++	++++	++++	++++	++++	++++	++++	+	+	+++	+
			24	+	+	+++	++++	++++	++++	++++	++++	++++	+	+	+++	+
Pat 73	-	STLV-1 Pat 74	0	-	-	-	-	-	-	-	-	-	-	-	-	-
			2	+	+	+	+++	++	+++	++	++	++	+	+	+	+
			4	+	+	+	+++	++	++++	++++	++++	++++	+	+	+++	+
			5	+	+	++	++++	++++	++++	++++	++++	++++	+	+	+++	+
			16	+	+	++	++++	++++	++++	++++	++++	++++	+	+	++++	+
Patt	-	STLV-1 Pat 74	0	-	-	-	-	-	-	-	-	-	-	-	-	-
			2	+	+	+	+++	+	++	+++	++	++	+	+	+	+
			4	+	+	+++	++++	++++	++++	++++	++++	++++	+	+	+++	+
			5	+	+	+++	++++	++++	++++	++++	++++	++++	+	+	+++	+
			16	+	+	+++	++++	++++	++++	++++	++++	++++	+	+	++++	+

* WB status = + if reactivity to both Gag p24 and Env gp46 original were observed, I + indeterminate, and – if no reactivity at all. The intensity of the WB was rated as ranging from negative (-) to 4 + .

▲ PCR was performed for both pol and pX genes. All + samples were + for both, and all – samples were – for both.

**Figure 2:**
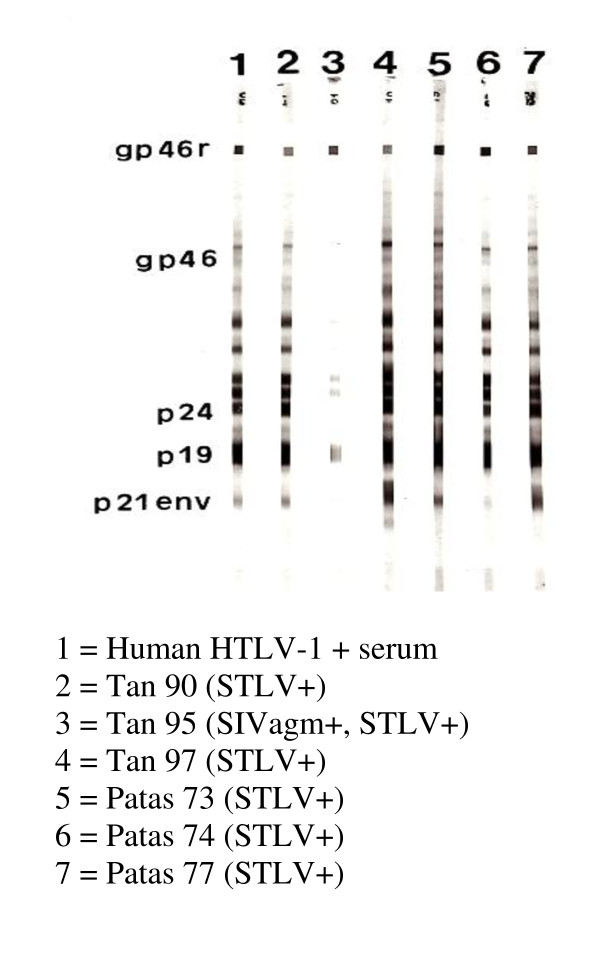
**Western blot profiles of various human and non-human primates infected with HTLV-I or STLV-I.** The Tan 95, 97, and Patas 73 and 77 samples were drawn two years post infection. A positive result is considered to be a reactivity to both p24 and gp46 or rgp46. All other reactivities are indeterminate.

**Figure 3:**
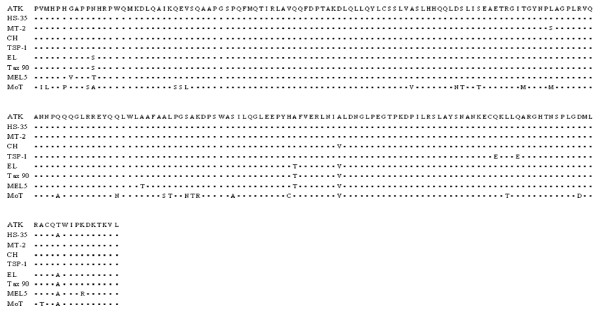
**Amino acid sequence alignment of the STLV-I (Tan 90) p24 Gag protein compared to various HTLV-I isolates and the HTLV-II (MoT) isolate.** The changes from the prototypic HTLV-I (ATK) isolate are shown.

Interestingly, while the *patas* monkeys had fully seroconverted by 2 months post-transfusion, both tantalus monkeys displayed prolonged seroconversion patterns. Both Tan 95 and Tan 97 took a year to become ELISA positive, and Tan 95 was still Western blot indeterminate at 2 yr post transfusion, never reacting to the Gag p24 protein (Table 1, Figure 2). In addition, the intensity of the WB reactivities of Tan 95 serum was much less than that of the sera from the other monkeys. Because STLV-1 Tan 90 is a relatively divergent African STLV-1 isolate, it was plausible that its p24 *gag* gene might be defective or that its cognate protein might be quite different from the Japanese HTLV-1 p24 antigen utilized in the Western blot [9-11]. Hence, we amplified, cloned and sequenced the p24 *gag* gene of STLV-1 Tan 90 (Figure 3). As can be seen, there are only minimal amino acid changes in the Tan 90 isolate relative to other PTLV-1 isolates.

In addition to their slower seroconversion to STLV-1, the target tantalus monkeys took longer than the *patas* monkeys to become PCR positive for STLV-1 DNA in their peripheral blood mononuclear cell (PBMC) (Table 1). When they did become STLV-1 positive, it was at a lower copy number (10) than the *patas* monkeys (100); although by 12 months, all infected monkeys stabilized at a viral load of 100 copies of STLV-1 DNA/μg PBMC DNA.

We decided to completely sequence STLV-1 Tan 90 (GenBank accession #AF074966), and partially sequence STLV-1 Pat 74 (GenBank accession # L20354.1) to ascertain whether sequence differences could explain the different STLV-1 viral loads and seroconversion rates observed in the recipient tantalus and *patas* monkeys. The complete LTR DNA sequences and deduced individual protein amino acid sequences of STLV-1 Tan 90 relative to HTLV-1 ATK are shown in Additional file 1. The organization of the LTR of both viral strains is identical. Complete U_3_, R, and U_5_ regions are identified. Within these regions there are no major differences in the poly (A) signal, TATA box promoter, distal, and proximal 21 bp enhancer regions, capsite, the sequences encoding the basic leucine zipper factor (bZ1P910), the EtS protein binding domain, the splice donor site, the Rex core site, and the primer binding site (PBS). The STLV-1 Pat 74 LTR sequence was similarly arranged and conserved (data not shown). Both HTLV-1 ATK and STLV-1 Tan 90 contained the same number and locations of putative methylation sites, 5’ to their promoters, but STLV-1 Pat 74 had two methylation sites (-291 and -60) altered from CpG to ApG which would render them methylase insensitive. Theoretically, this would make STLV-1 Pat 74 less susceptible to down modulation of viral RNA transcription by DNA methylation. Relative to HTLV-1 ATK, there were changes in both the STLV-1 Tan 90 and STLV-1 Pat 74 middle 21 bp repeat enhancer sequences. The former had a G-A transition outside of any consensus DNA protein binding domain, while the latter had an A-G transition in domain A (AP-2 consensus site). The effects of these changes are unknown. Throughout their genomes, consensus splice donor and splice acceptor sites found in HTLV-1 ATK were conserved in both STLV-1 Tan 90 and STLV-1 Pat 74 (data not shown).

**Figure 4:**
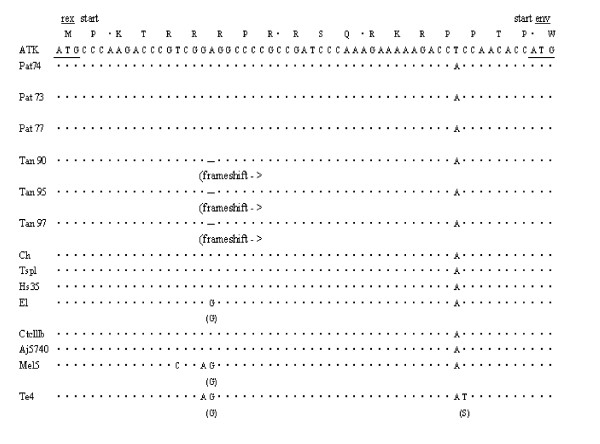
**Alignment of partial nucleotide sequences of the *rex *gene for all *patas *and tantalus STLV-1 strains used in this study compared to other different HTLV-1 strains.** The base deletions at position 5164 (ATK EMBL#) fro the tantalus strains, respectively, are shown as dashes which would create a frameshift and allow for missense translation of p27 Rex accordingly. p21 Rex would not be affected because it utilizes a translational start site downstream from the deletions. ATK is the consensus Japanese HTLV-1 strain used for comparative analysis with its translated amino acids displayed above each codon. Areas of identity are indicated by the symbol (•) and deletions shown by the symbol (―). Letters displayed in parenthesis below a sequence represent an alteration in an amino acid codon at that position. For clarity, the missense translations of the STLVs are not shown. The alignment was ended at the 5’ splice junction for *rex* located in the *env* gene of the viral genome.

As can be seen (Additional file 1), there are minimal amino acid differences between the deduced prototypic HTLV-1 ATK and STLV-1 Tan 90 Gag, Protease, Env, Tax, p21 Rex and P13^II^ proteins. The stop codon in the ATK Pro is probably a sequencing error because this is not observed in any other PTLV-1 strain. It is difficult to know whether any of these minor changes could effect STLV-1 Tan 90 replication. There are greater amino acid differences in the p30^II^ and p12^I^ proteins, but again it is unclear how much this would affect their function. However, a C to T base substitution at position 2560 in the STLV-1 Tan 90 *pol* gene creates an early stop codon, and a deletion of an A at position 5140 in the *pol* gene eliminates a terminal stop codon present in the HTLV-1 ATK protein (Additional file 1 and Figure 4). This same deletion also causes an early frameshift in the p27 Rex protein, such that it is initially translated in the Tax reading frame, and then, because of splicing, results in a nonsense sequence (Additional file 1 and Figure 4). None of these changes are present in the STLV-1 Pat 74 sequence, nor in any other published PTLV-1 sequence (Additional file 1 and Figure 4). However, both mutations are present in the STLV-1 Tan 95 and 97 sequences (Additional file 1 and Figure 4), indicating that, while they may affect the replication rate of STLV-1 Tan 90, they did not prevent its transmission to other monkeys.

## Discussion

Over presumably tens of thousands of years the PTLV have been disseminated among human and non-human primates throughout the planet. While phylogenetic data indicate that this dissemination has been predominantly intraspecies, evidence for episodic interspecies transmission exits as well [10,12-14]. It is unclear whether the replication rate or the pathogenicity of a particular substrain of the PTLV will be the same in all primates. In an effort to study the PTLV further, we established non-human primate models starting with the intraspecies transmission of two highly divergent isolates, STLV-1 Tan 90 and STLV-1 Pat 74. The data presented herein, indicate that successful infection can be achieved by transfusing whole blood from infected tantalus and *patas* monkeys to target animals of the same species. As anticipated, there was no evidence of genetic drift in the STLV-1 *pol* and *pX* sequences analyzed between the original isolates and those found in the PBMC of the target animals. Also, none of the animals developed overt clinical disease during the 2–3 year followup period.

Interestingly, the Tan 95 and Tan 97 monkeys had prolonged seroconversion rates and different patterns compared to the Pat 73 and Pat 77 monkeys. It is doubtful that the PCR analyses, which confirmed infection in the tantalus monkeys, are false positives because they were performed three times and considerable effort was made to prevent and detect PCR “carryover”. Also the STLV-1 sequences characterized in these two target monkeys are identical to the unique sequences of the STLV-1 Tan 90 strain with which they were inoculated. Finally, both monkeys eventually made antibodies to the HTLV-1 antigens utilized in the ELISA and Western blot assays, albeit Tan 95 never made detectable antibodies to the HTLV-I p24.

The diminished antibody response to the HTLV-I p24 antigen by Tan 95 could be explained if the STLV-1 Tan 90 isolate’s p24 Gag antigen was highly divergent form prototype HTLV-1, but, in fact, it is not (Figure 3). This is consistent with the fact that the original tantalus monkey infected with STLV-1 Tan 90 was positive in both the ELISA and WB assays used herein [9,10]. Also, it has been well established that many conserved seroreactive epitopes exist among the PTLV and even bovine leukemia virus p24 Gag antigens [3,10-12,15,16].

It is doubtful that the difference between the seroconversion patterns between the tantalus and *patas* monkeys was due to quantitatively different innocula of STLV-1. However, the initial levels of STLV-1 DNA in the experimentally infected Tan 95 and Tan 97 monkeys were very low relative to those of the target *patas* monkeys, suggesting that a lower replication rate was the cause of the slower seroconversion.

It is difficult to say if the pre-existing SIV infection in Tan 95 had anything to do with its aberrant seroconversion. SIV is not felt to be pathogenic in African green monkeys and, by all analyses, Tan 95 was healthy and immunocompetent [17]. Delayed seroconversion to PTLV infection has been described before for both HTLV-1 and HTLV-2 naturally infected humans, and STLV-1 naturally infected non-human primates [3,4,10]. This phenomenon probably has more to do with the rate and pattern of PTLV protein expression than with the immune status of the host. Possible explanations for the inefficient expression of HTLV-1 proteins have included the presence of defective proviral DNA, the low rate of production of singly spliced and unspliced viral mRNA and the presence of natural antisense viral RNA [13,18]. While other changes such as differences in LTR methylation sites and the middle 21 bp enhancer sequence may play a role, it would seem most likely that the stop codon mutation in the *pol* gene found in STLV-1 Tan 90 is a major reason for the slow seroconversion rates observed in Tan 95 and Tan 97. The fact that STLV-1 Tan 90 was transmitted to both Tan 95 and Tan 97 indicates that this mutation is not lethal. However, it would presumably require either one or more molecular events, such as read through termination suppression or using a downstream AUG translational start site, for functional STLV-1 Tan 90 Pol proteins to be produced [19]. Such a downstream start site is present not too far from the amino terminus of STLV-1 Tan 90 *pol* RNA (Additional file 1) While the predicted large Pol protein, due to the deletion at position 5140, might still produce a functional integrase protein, it is possible that proteolytic activity could cleave it into a smaller molecule [20].

The changes observed in the p27 Rex protein of STLV-1 Tan 90 would seemingly render it non-functional. Because Rex regulates expression of unspliced PTLV-1 RNAs, it would seem obvious that the STLV-1 Tan 90 would replicate more slowly in the host relative to other published PTLV strains, including STLV-1 Pat 74 [21]. In particular, a non-functional p27 Rex protein would result in less Gag p24 expression. Our data would indicate that a fully functional p27 Rex protein is not absolutely required for in vivo STLV-1 transmission. Whether p21 Rex could partially replace p27 Rex is unclear, but certainly possible; although it localizes to the cytoplasm rather than the nucleus [22,23]. Another possible “rescue” mechanism for translation of the STLV-1 Tan 90 p27 Rex protein could be a ribosomal frameshift [24,25]. Retroviral frameshifting usually occurs on an slippery heptanucleotide of the form XXXYYYZ. Two tRNAs bound to nucleotides 2 to 7 of this site simultaneously slip 1 to 2 bases leftward onto nucleotides 1 to 6 stimulated in part by a secondary structure in the downstream mRNA molecule called a pseudoknot. While the STLV-1 Tan 90 Rex RNA sequence does not have a “slippery” heptanucleotide at the site of the initial frameshift mutation, it does have a CCCAAAG heptanucleotide not too far downstream (Figure 4). There is also an alternative splice site just downstream from the deletion in the STLV-1 Tan 90 *pol/rex* genes. If utilized, instead of the routine splice site, an in-frame p27 Rex protein with a variable amino terminus would be produced.

An interesting question is how frequent the mutations observed in STLV-1 Tan 90 occur in other wild-type PTLV-1 strains and what their clinical implications might be. While it might seem intuitive that a slower replicating PTLV-1 strain might be less oncogenic, others have postulated that repression of virus expression, particularly by p30^II^, might allow for evasion of immunodestruction of virus infected cells and a higher probability of T-lymphocytic transformation [26]. Further, epidemiological studies would seem warranted to answer the above questions.

## Methods

### Animal trapping, infection and sampling

Wild *C.* tantalus and *E. patas* monkeys were captured in Central African Republic. Approval for collecting simian specimens was granted by the Comité Consultatif D’Ethique en Experimtation Animale (C.C.E.E.A.) de l’Ecole Inter-Etats des Sciences et Medicine Veterinaires de Dakur. As previously described, two of these monkeys, Tan 90 and Pat 74, were found to be infected with the unique STLV-1 strains Tan 90 and Pat 74 [9,10], respectively. Two tantalus monkeys (Tan 95 and 97) and 2 *patas* monkeys (Pat 73 and 77) were found to be consistently negative for STLV-1 by serological, PCR and virus culture analyses [10]. One of these monkeys, Tan 95, was found to be infected with the simian immunodeficiency virus (SIV) [17]. All of the monkeys were deemed to be healthy without signs of leukemia nor immunodeficiency. All had normal CD4 counts (range 660-1200/mm^3^), CD8 counts (range 100–500 cells/mm^2^), and immunoglobulin levels (eg IgG range 1000–3000 mg/dL). These animals were tested for STLV three times in the months prior to inoculation including the day of inoculation.

Three ml of whole blood collected in EDTA from Tan 90 were transfused into both Tan 95 and Tan 97, and an equivalent amount of whole blood from Pat 74 was transfused into Pat 73 and Pat 77. Quantitative DNA PCR indicated that the STLV-1 viral load in these inocula were approximately the same (100 copies of STLV-1 DNA per μg of primate DNA). The experimentally and naturally infected monkeys were monitored for an additional two years with routine physical exams, examination of their complete blood count, differential CD4 and CD8 counts, and immunoglobulin levels. Periodically, aliquots of their heparinized whole blood were separated into plasma and PBMC and examined for STLV-1 antibodies and DNA, respectively.

### Serological assays

Plasma were analyzed using an HTLV-1 whole viral antigen ELISA (Diagnostic Biotechnology Singapore) and a Western blot kit (Diagnostic Biotechnology), which in addition to HTLV-1 whole viral antigens, also includes recombinant HTLV-1 rgp21 and rgp46 Env peptides [11]. US Public Health criteria were used to designate a serologic result as positive, negative or indertminate [27].

### PCR

One μg of organically extracted DNA from the monkey PBMC was amplified and detected via PCR using the PTLV 1/2 generic *pol* (SK110/SK111) and *tax* (SK43/SK44) primers, and the HTLV-1/STLV-1 specific detector SK112 and the PTLV 1/2 generic detector SK45, as previously described [12]. All samples were also analyzed for primate β-globin DNA as previously described to assure that amplifiable DNA was present in the sample [12]. All PCR assays were done in triplicate. In order to avoid false positives due to “carryover” of previously amplified DNA, all pre- and post- PCR steps were conducted in completely different facilities by different personnel. In addition, all amplifications of the above regions done in our laboratory utilize dUTP rather than TTP, and all amplifications were subjected to PCR sterilization with uracil glycosylase [28]. Finally, all primers contain 5’ non-viral non-primate linker sequences, which, in addition to facilitating the cloning of amplified DNA (see below), allow for the use of “signature primers” for the detection of “carryover” DNA. Accordingly, all STLV-1 PCR positive samples were reanalyzed with “signature primers” and found to be negative for “carryover” DNA [29]. Quantification was estimated by comparison of hybridization signals to a serially diluted positive control, and also by serially diluting the input DNA sample and calculation of the Poisson distribution in the original sample. This assay is 100% and 58% sensitive down to concentrations of 10 copies and 1 copy per aliquot, respectively.

To examine the *gag* p24 (bases 1214–1855 as per the EMBL numbering system) region of the STLV-1 strains, the following overlapping primers were utilized: HTIL (715–734) + (TACTGGCTCGGAGCCAGCAG); HTIG (1499–1479) – (GACCGGCTAAGGGGTTATAAC); HTIG (1423–1444) + (CCATCACCAGCAGCTAGATAGC); AND HTIG (1919–1899) – (AGTGGCCTGCTTTCCCGCACC). The probe utilized was HTIG (1475–1507) + d (ACAGGTTATAACCCATTAGCCGGTCCCCTCCGT). The bases are all listed 5’ to 3’.

### Cloning and sequencing

**Figure 5:**
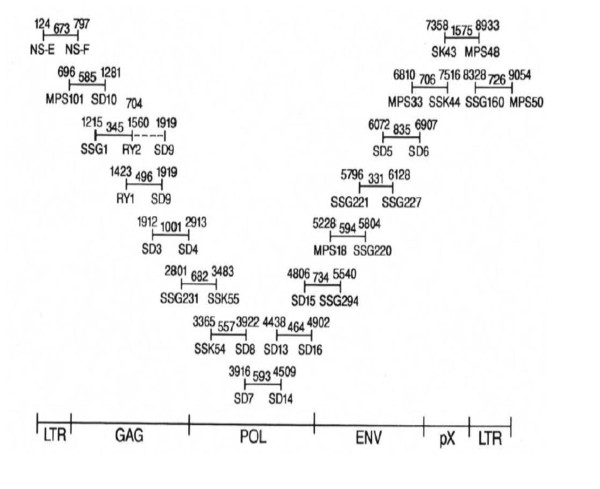
**Schematic representation of overlapping PCR primer pairs utilized to amplify the entire STLV-1 genome of strain Tan 90.** The numbers above the primer notations are according to their position in the EMBL consensus HTLV-I sequence while the length of the fragment contributed by each primer pair is shown in between. The DNA amplicons produced were cloned and sequenced to further investigate the molecular identity of STLV-I Tan 90.

Cloning of the *p24*, *pol* and *pX* PCR amplified products, listed above, was accomplished by digesting the DNA with Kpn I and SstI, and subsequent ligation into M13mp18. The ligation mixes were used to transfect competent KT8052 (ung-) *E. Coli* cells plated with media containing 5-bromo-4chloro-33indolyl B-D galactoside and isopropyl B-D thiogalactoside [10,30]. Positive clones were detected by plaque hybridization with the appropriate specific oligonucleotide probe (see above) and the DNA from the positive plaques was sequenced by the dideoxynucleotide chain termination method [31]. The full length sequence of STLV-1 Tan 90 and the partial sequence of STLV-1 Pat 74 were derived using a series of overlapping PCR primers, probes, cloning and sequencing, as previously described (Figure 5) [9]. Sequencing was performed in both directions and all major changes were verified in at least five clones.

Nucleotide sequence alignments were generated using a commercial software package [32].

## Competing interests

The authors declare that they have no competing interests.

## Authors’ contributions

SD, NS, TS, JH, and PB participated in molecular studies. NS was involved in the serologic studies. SD, DD and BP participated in the sequence alignment and analysis. NS and BP conceived of the study. NS, SD, DD and BP participated in the design and coordination of the study and drafting of the manuscript. All authors read and approved the final manuscript.

## Supplementary Material

Additional file 1 **Nucleic acid (LTR) and amino acid sequences of HTLV-1 ATK and STLV-1 Tan 90.** Areas of homology are indicated by the symbol (•), deletions by (-) and stop codons by (*). The various base or amino acid substitutions are as indicated. In the LTR the junctions between U3, R and U5 are shown, the three 21 bp repeat enhancer sequences are underlined, the primer binding site (PBS) is overlined. The basic leucine zipper factor (bZ1P910), poly A signal, TATA box promoter (AP site, splice donor (SD) and *rex* core sites are labeled. Functional areas of the PTLV-1 Tax primer are identified. Click here for file

## Sequence data

GenBank: AF074966.1; L20354.1
