# Supplementary figures and images for: Delayed seroconversion to STLV-1 infection is associated with mutations in the pol and rex genes
Source: Virol J. 2013 Sep 11;10:282. doi: 10.1186/1743-422X-10-282 (PMC3851238; doi:10.1186/1743-422X-10-282)

**LTR**
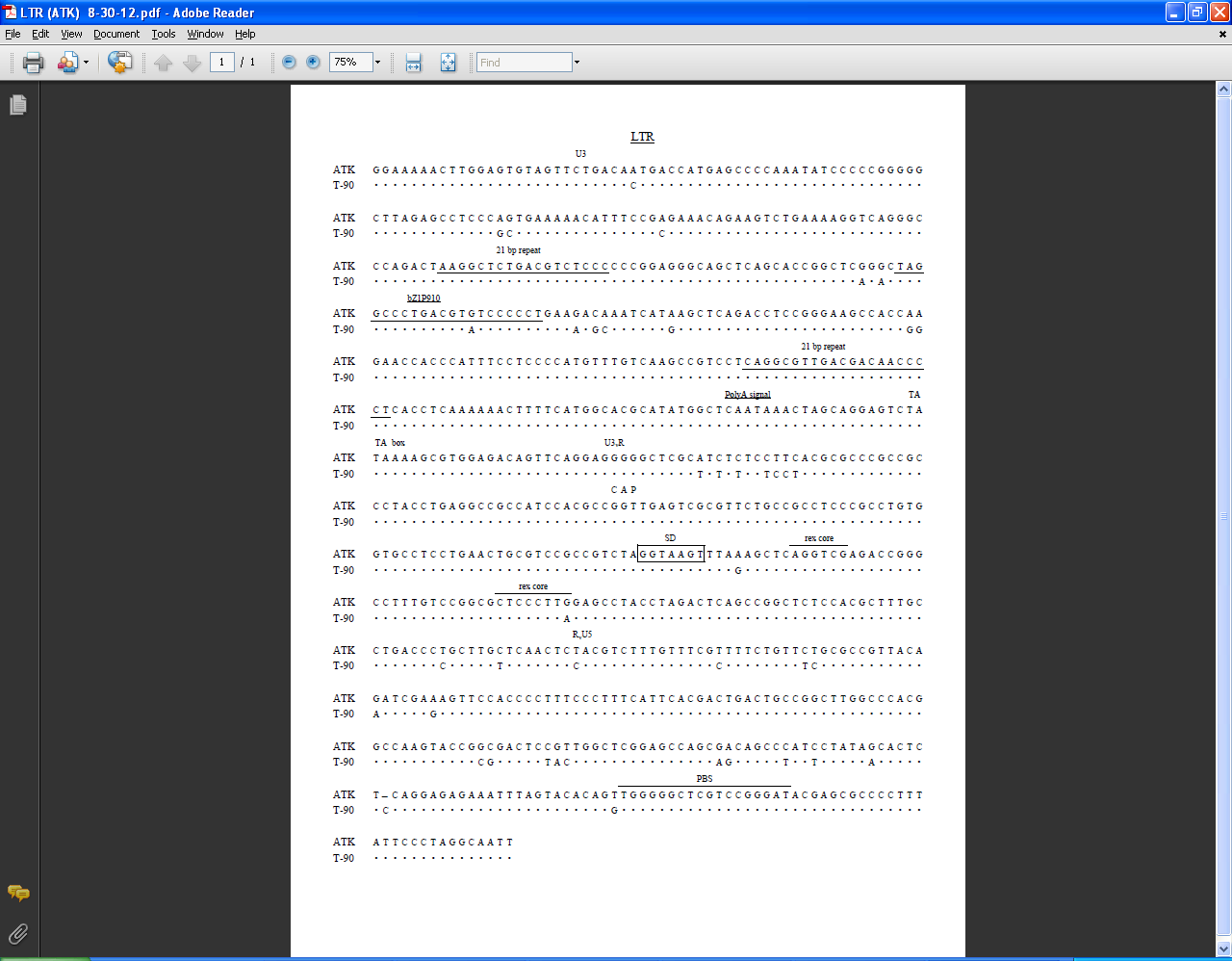


**gag**


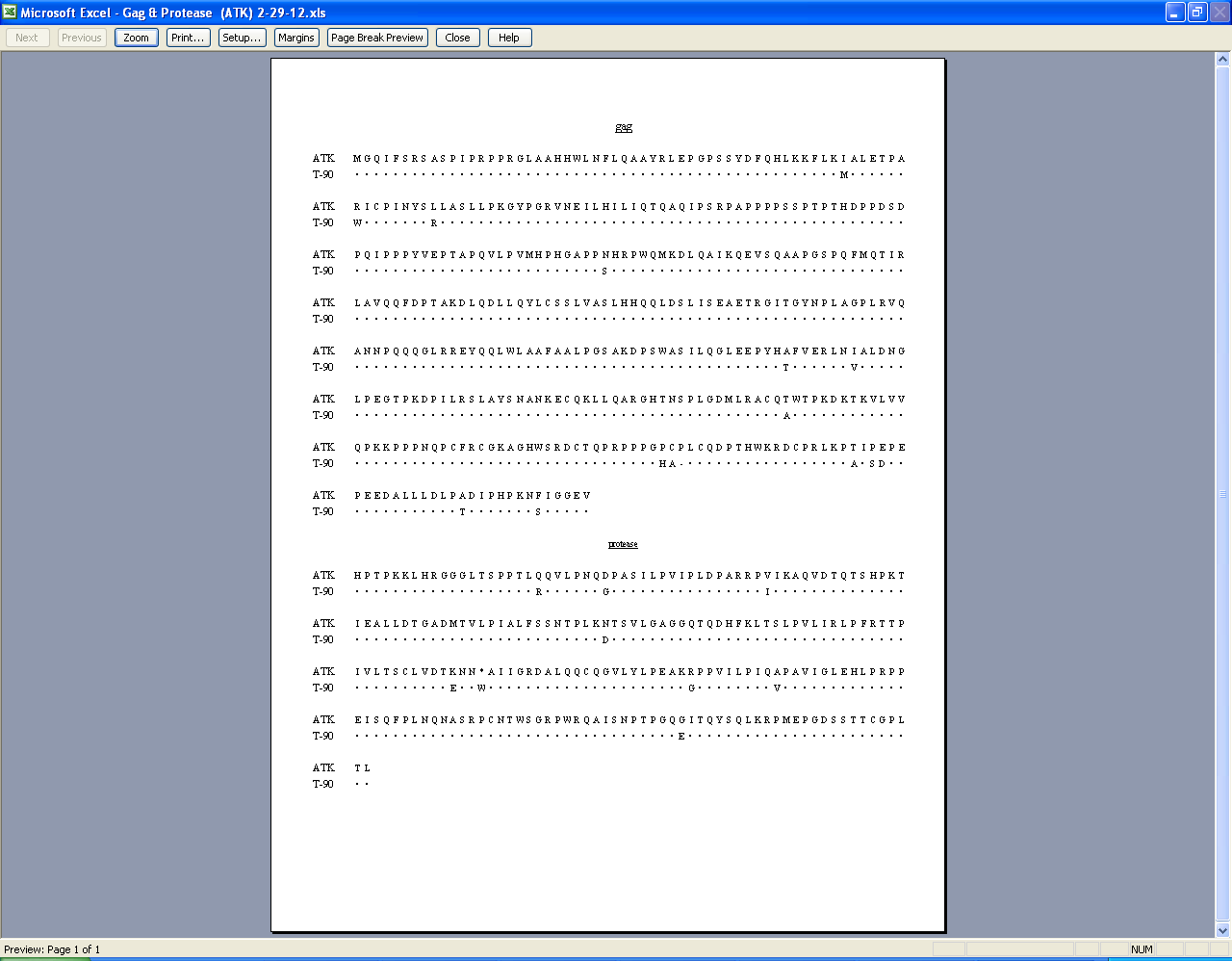


**Protease**


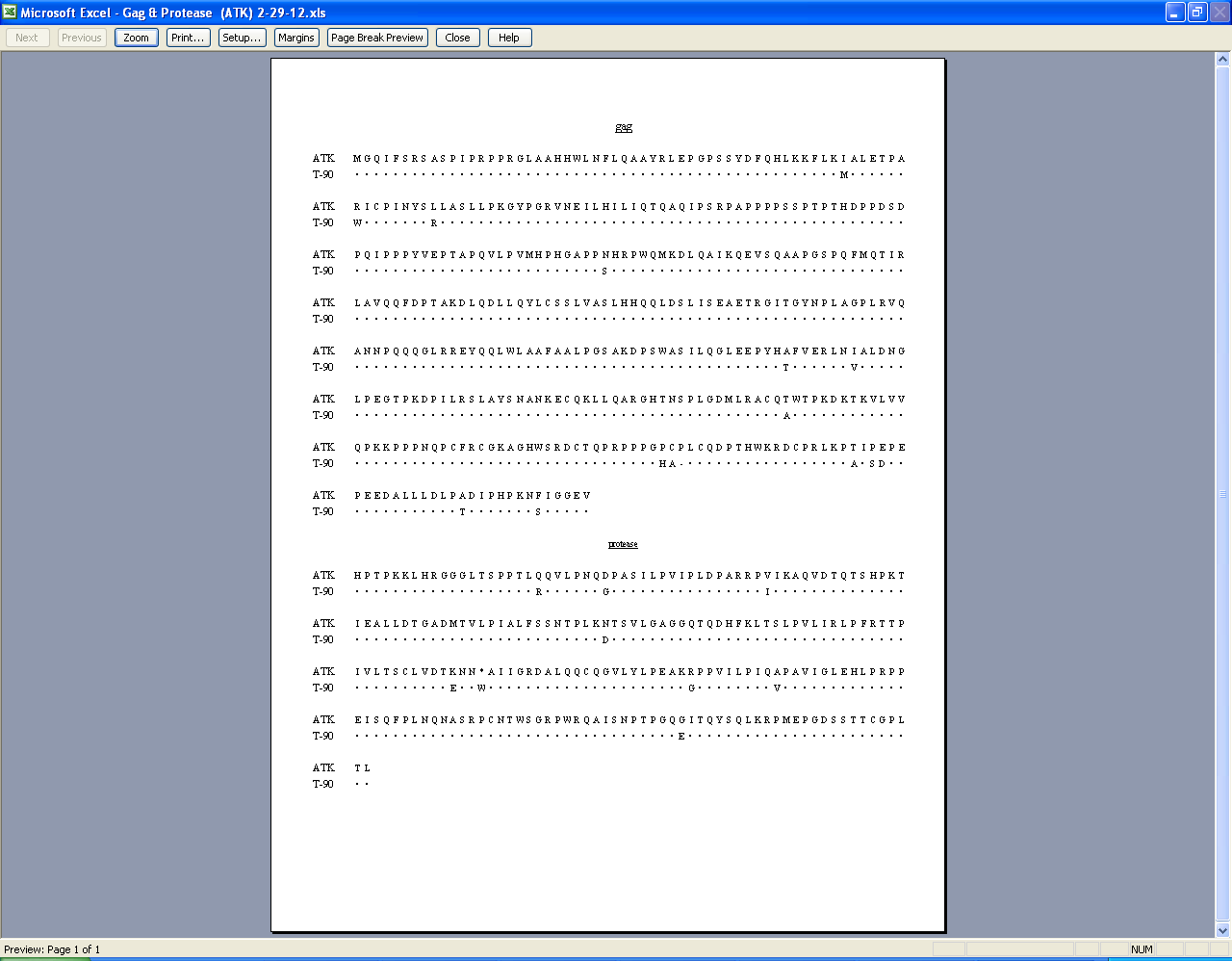


**pol**


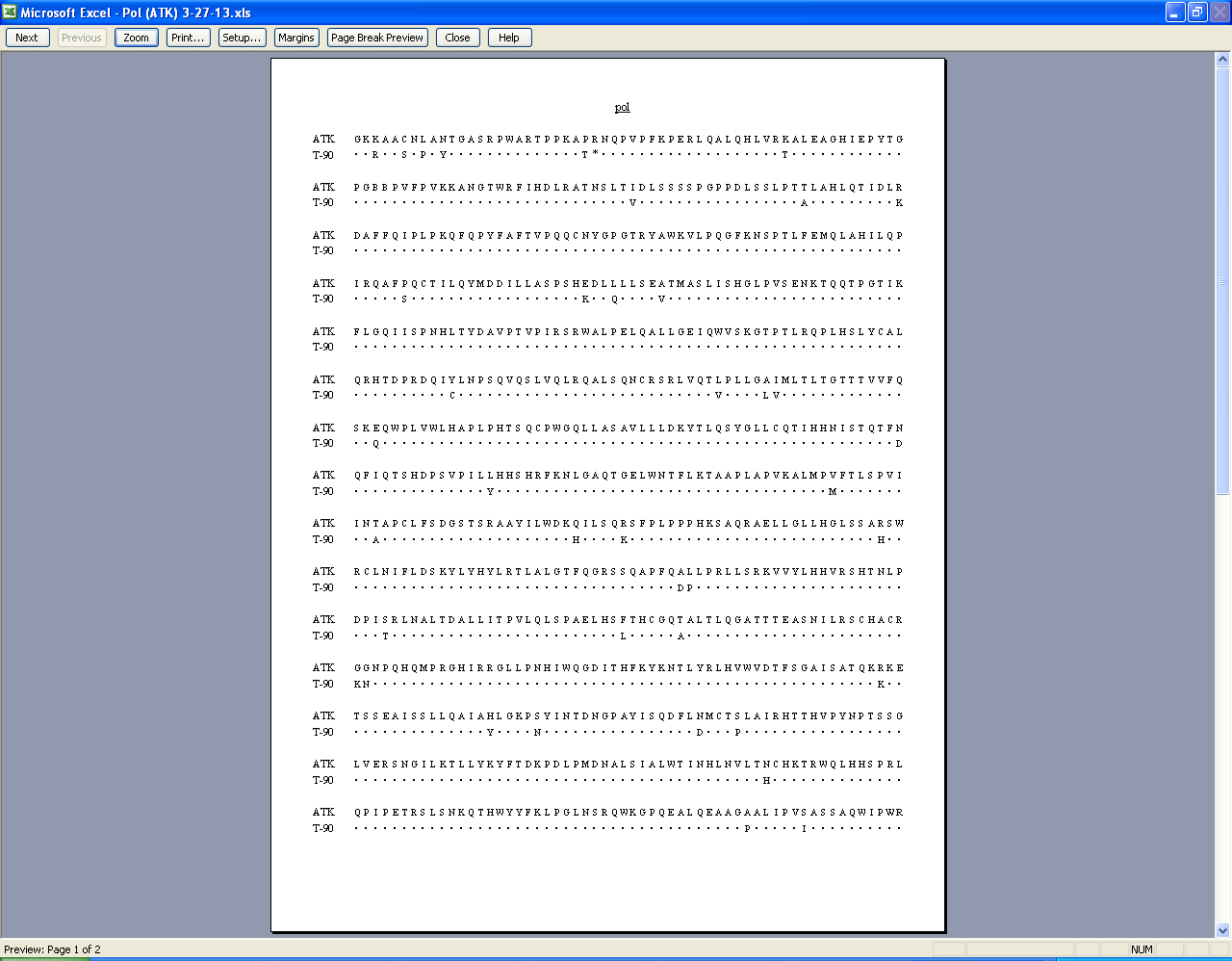


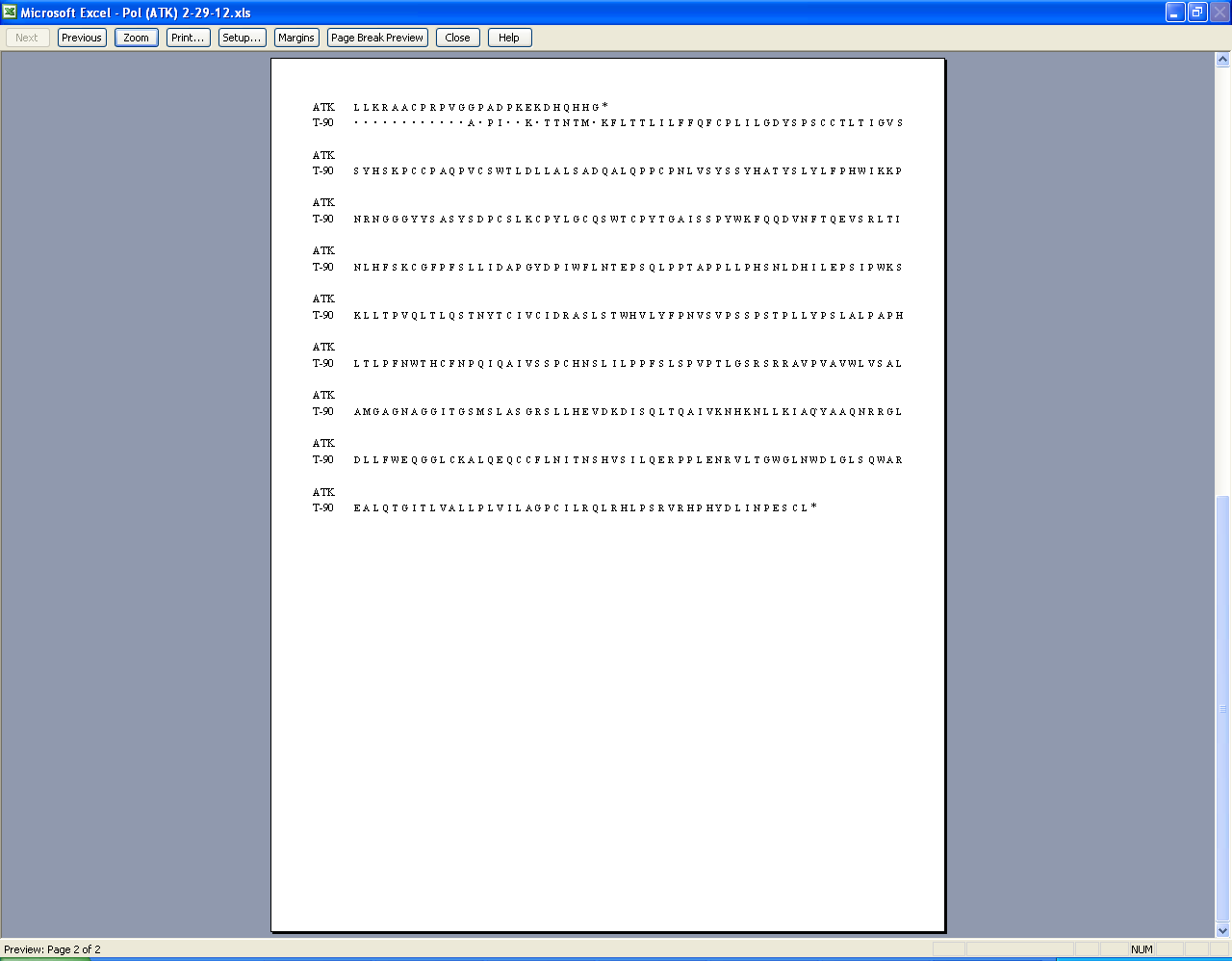


**env**


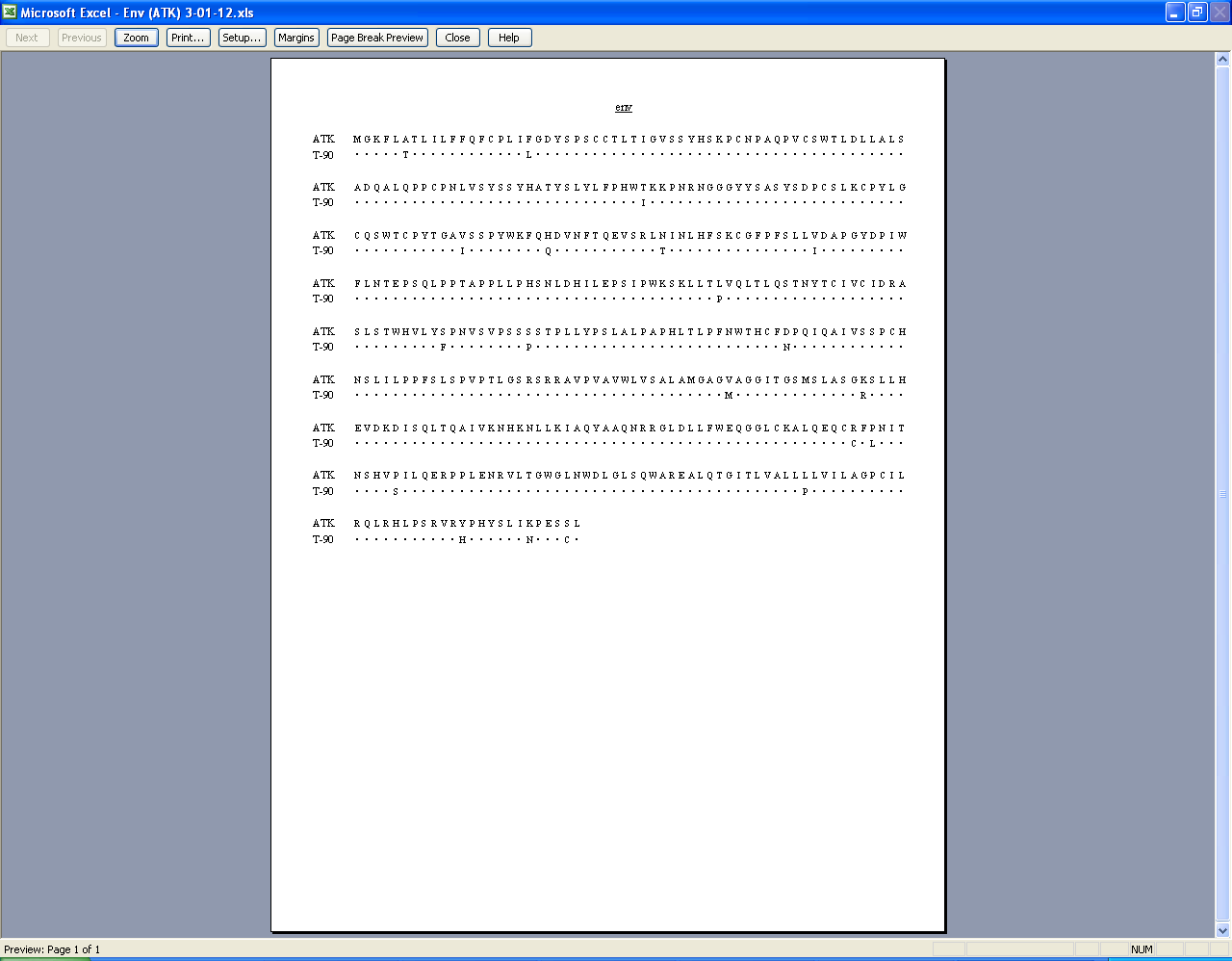


**tax**


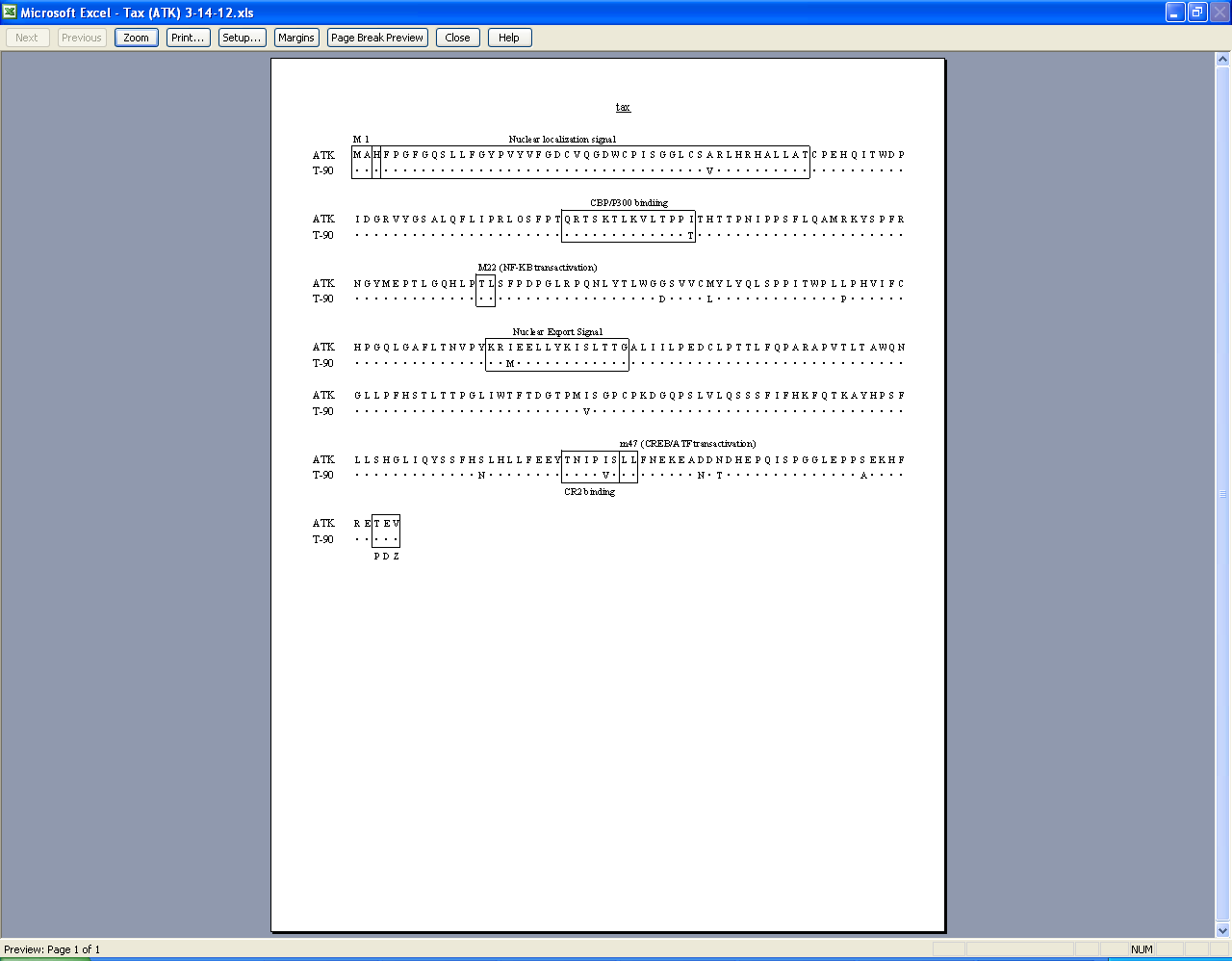


**p27 Rex**


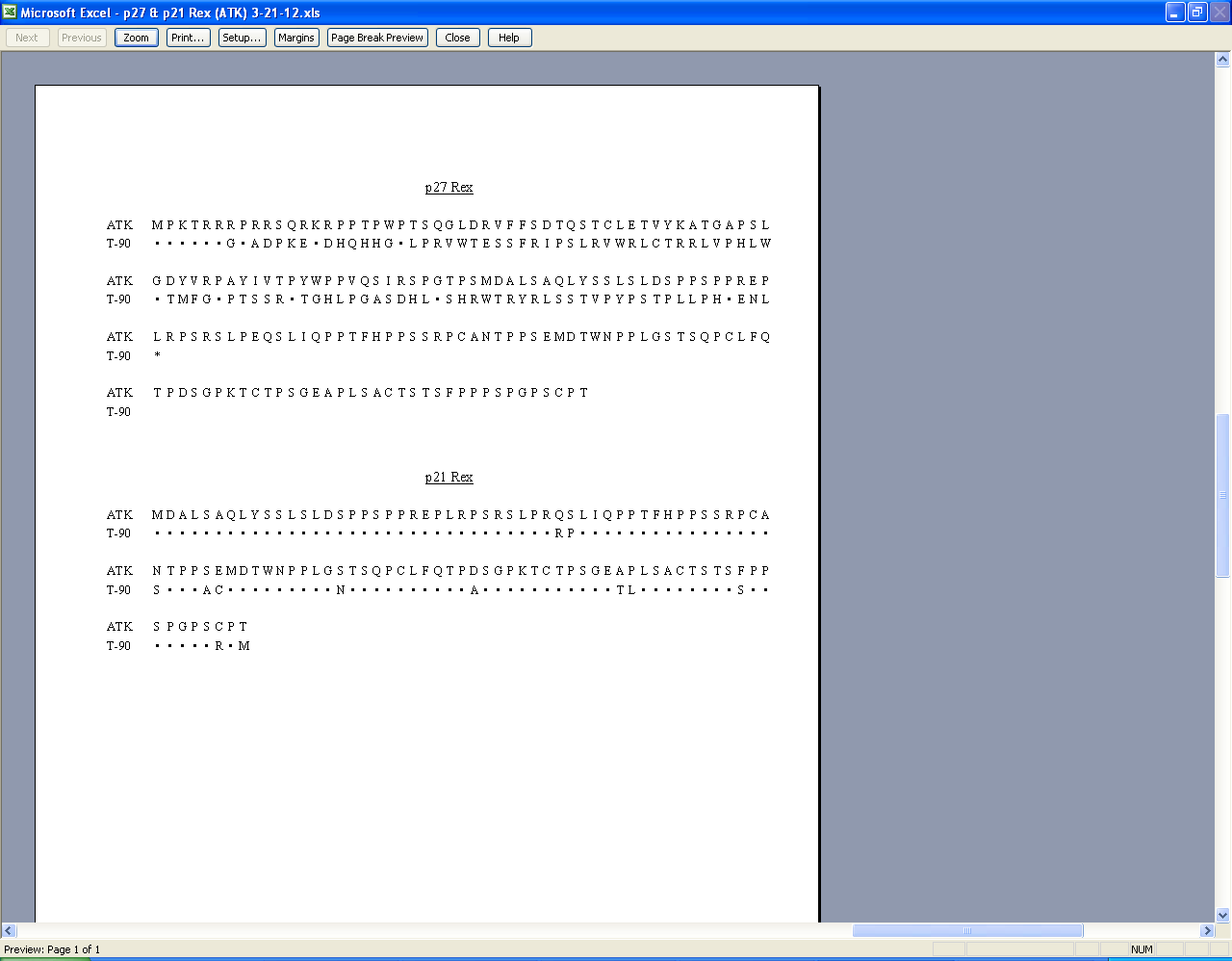


**p21 Rex**


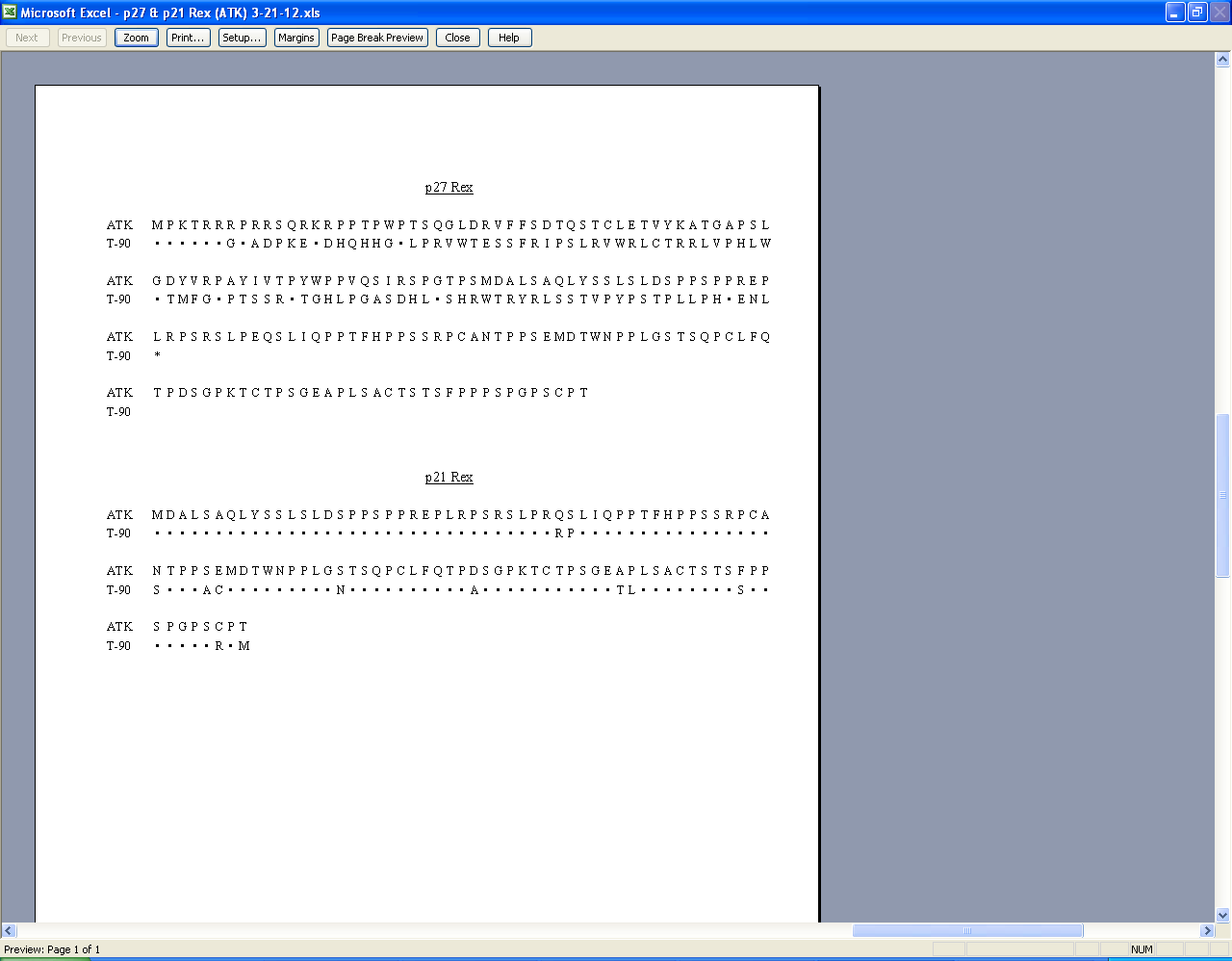


**p13II**


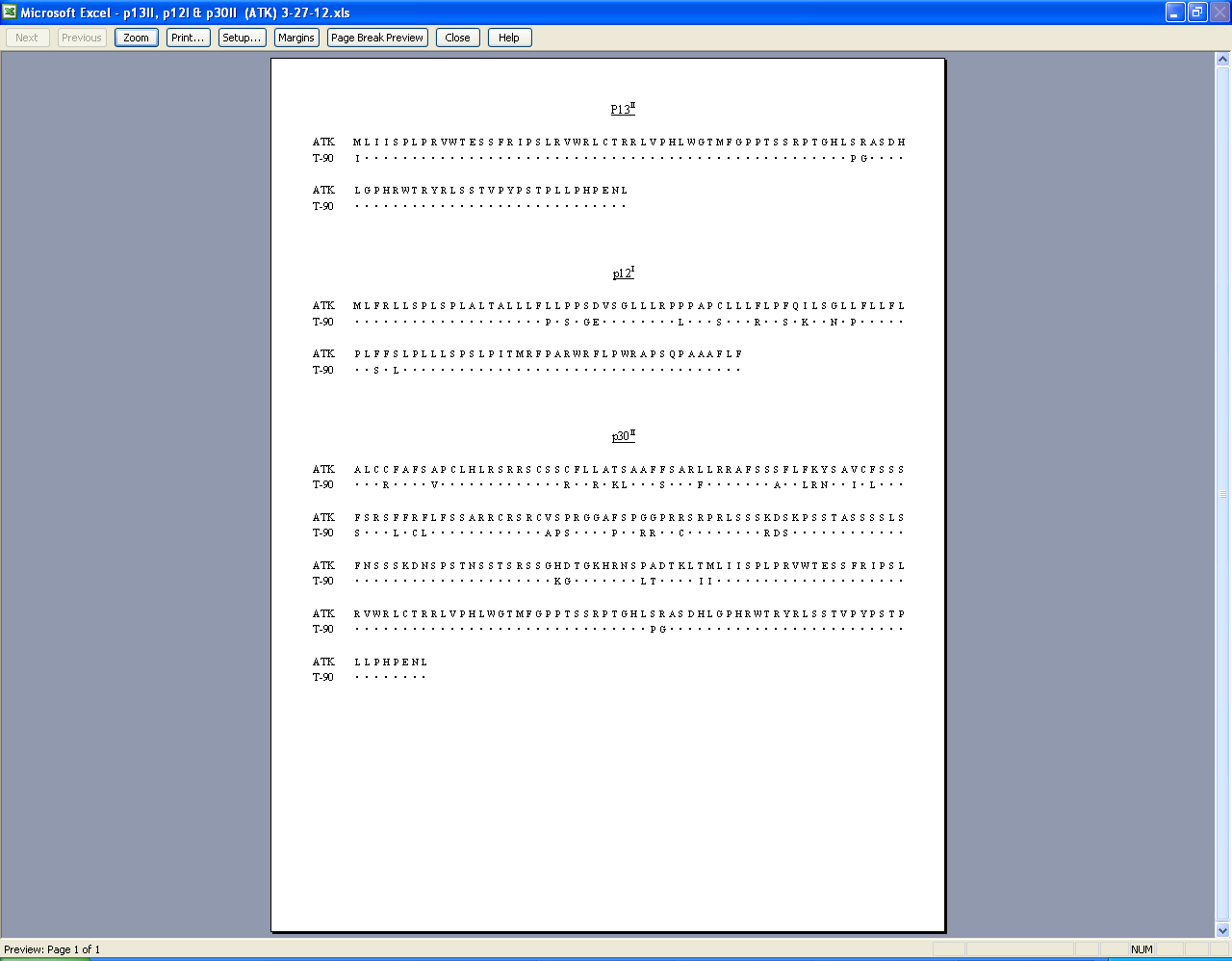


**p12I**


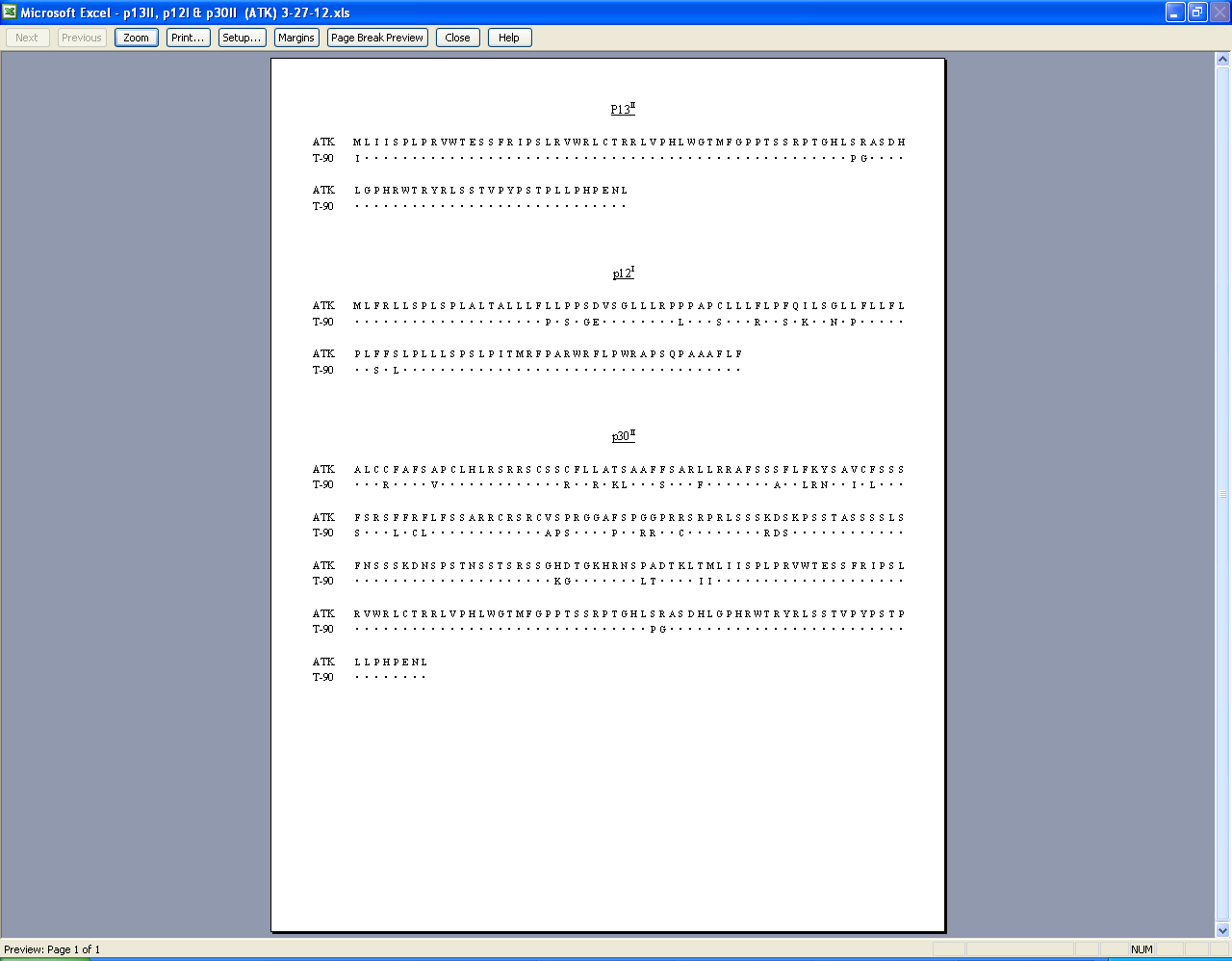


**p30II**


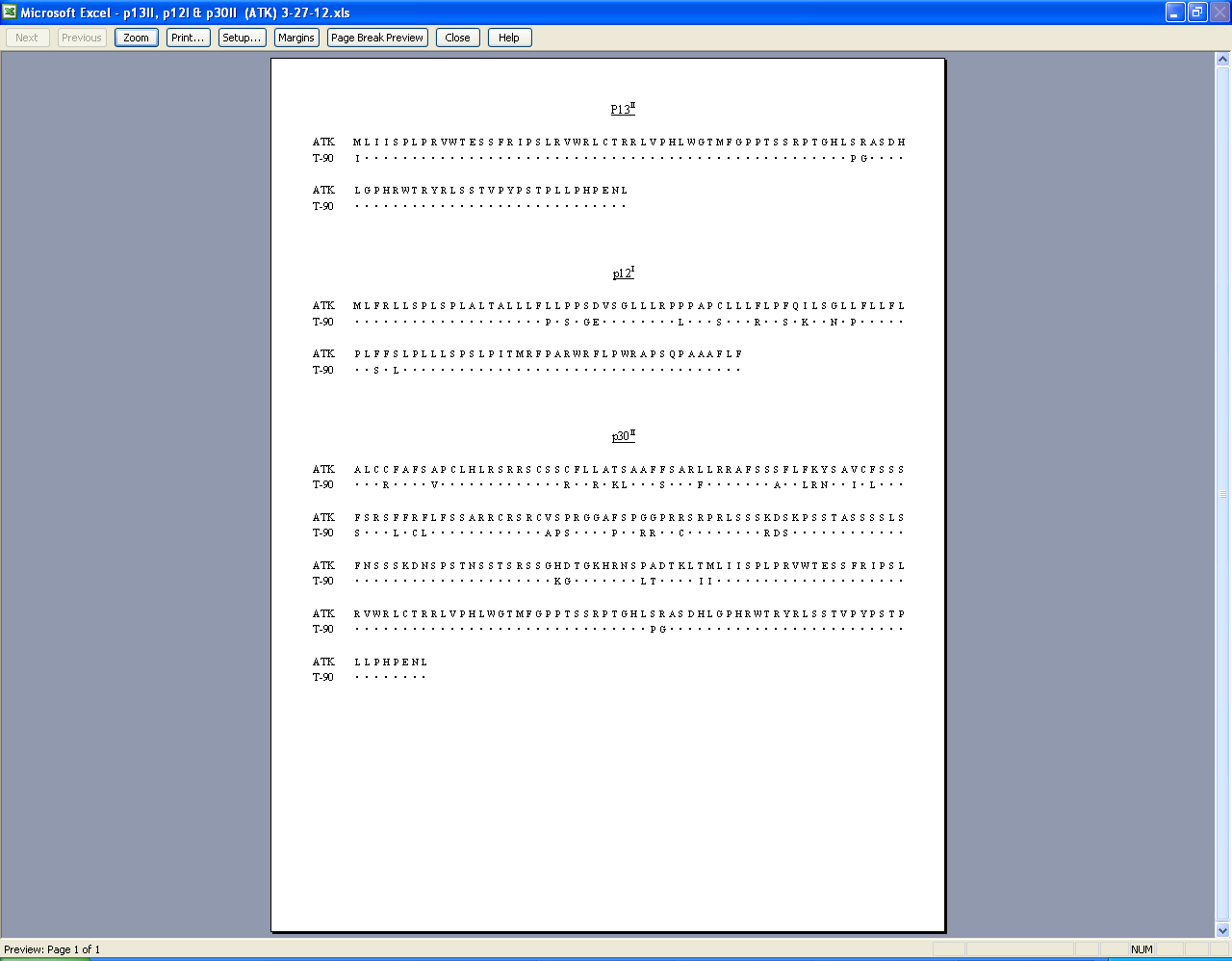

Supplement: Additional file 1 — Nucleic acid (LTR) and amino acid sequences of HTLV-1 ATK and STLV-1 Tan 90. Areas of homology are indicated by the symbol (•), deletions by (-) and stop codons by (*). The various base or amino acid substitutions are as indicated. In the LTR the junctions between U3, R and U5 are shown, the three 21 bp repeat enhancer sequences are underlined, the primer binding site (PBS) is overlined. The basic leucine zipper factor (bZ1P910), poly A signal, TATA box promoter (AP site, splice donor (SD) and rex core sites are labeled. Functional areas of the PTLV-1 Tax primer are identified. [file 1743-422X-10-282-S1.docx]
